# Enhanced Piezo-Photocatalytic Performance of Na_0.5_Bi_4.5_Ti_4_O_15_ by High-Voltage Poling

**DOI:** 10.3390/ma16145122

**Published:** 2023-07-20

**Authors:** Shuang Lan, Mupeng Zheng, Fangping Zhuo, Mankang Zhu, Yudong Hou

**Affiliations:** 1 Key Laboratory of Advanced Functional Materials, Faculty of Materials and Manufacturing, Beijing University of Technology, Ministry of Education, Beijing 100124, China; 2 Department of Materials and Earth Sciences, Technical University of Darmstadt, 64287 Darmstadt, Germany

**Keywords:** piezo-photocatalysis, Na_0.5_Bi_4.5_Ti_4_O_15_, poling process, piezoelectric effect

## Abstract

The internal electric field within a piezoelectric material can effectively inhibit the recombination of photogenerated electron–hole pairs, thus serving as a means to enhance photocatalytic efficiency. Herein, we synthesized a Na_0.5_Bi_4.5_Ti_4_O_15_ (NBT) catalyst by the hydrothermal method and optimized its catalytic performance by simple high-voltage poling. When applying light and mechanical stirring on a 2 kV mm^−1^ poled NBT sample, almost 100% of Rhodamine B solution could be degraded in 120 min, and the reaction rate constant reached as high as 28.36 × 10^−3^ min^−1^, which was 4.2 times higher than that of the unpoled NBT sample. The enhanced piezo-photocatalytic activity is attributed to the poling-enhanced internal electric field, which facilitates the efficient separation and transfer of photogenerated carriers. Our work provides a new option and idea for the development of piezo-photocatalysts for environmental remediation and pollutant treatment.

## 1. Introduction

Photocatalytic technology has been widely studied in recent years due to its low cost, wide energy source, and no secondary pollution, making it a promising approach for water pollution treatment [1,2,3]. However, the slow migration and rapid recombination of photogenerated carriers seriously restrict the development of photocatalytic technology [4,5,6,7]. In order to improve the separation efficiency of photogenerated carriers, researchers have carried out a lot of studies on adjusting the energy band structure [8], doping [9,10,11], metal deposition [12,13,14], and the formation of heterostructures [15,16]. Despite these efforts, the challenge of achieving high quantum efficiency in photocatalytic systems still remains.

Recently, there has been a growing interest in harnessing the built-in electric field generated by piezoelectric/ferroelectric materials to enhance photocatalytic efficiency [17,18,19,20]. Piezoelectric/ferroelectric materials subjected to external forces will cause displacement of positive and negative charge centers, thereby generating a built-in electric field [21,22,23]. The existence of a built-in electric field can not only modulate the energy band structure of the photocatalyst, but also provide a driving force for the migration and separation of photogenerated electrons and holes, which is conducive to the redox reaction [24,25].

As a layered bismuth-based piezoelectric material, Na_0.5_Bi_4.5_Ti_4_O_15_ (NBT) has a unique (Bi_2_O_2_)^2+^-layer and anion-layer staggered charged-layer structure [26,27], in which the formation of Bi-O bonds will cut the anion layer, resulting in the distortion of TiO_6_ octahedron and then spontaneous polarization [28,29]. In addition, oxygen vacancies are easily generated in the (Bi_2_O_2_)^2+^ layer, expanding the wavelength coverage of light response [30]. The presence of these properties is believed to easily form a built-in electric field, which effectively promotes charge separation and enhances photocatalytic activity [31,32]. However, since the intrinsic piezoelectric coefficient of NBT is too low, and can only respond in the ultraviolet region, the piezo-photocatalytic performance of NBT still needs to be improved. Recently, our study has shown that by modifying Cu metal particles on the surface of NBT piezoelectric particles, the piezo-photocatalytic performance of NBT can be significantly improved [33]. The rate constant *k*_obs_ value is ~13 times larger than that of the pure NBT, indicating that the catalytic performance of NBT has great room for improvement.

The generation of a built-in electric field comes from the directional arrangement of domains in ferroelectric materials, which in turn affects the polarization intensity of materials. When domains are randomly arranged, the dipole moments cancel each other out, leading to a low polarization intensity. Conversely, when domains are aligned in a more uniform manner, the enhanced polarization intensity provides an additional driving force for the separation of photogenerated carriers, thereby improving the overall photocatalytic performance [34]. Previous studies have demonstrated that the applied poling electric field can promote the domain arrangement to be consistent and enhance the internal polarization field, thus improving and prolonging the separation efficiency and lifetime of the photogenerated carriers. For instance, Li et al. exerted corona poling on Bi_2_MoO_6_ ultrathin nanosheets, which exhibited significant CO_2_ reduction activity far exceeding that of unpoled Bi_2_MoO_6_ [35]. Huang et al. prepared poled BiFeO_3_ nanoparticles by electric poling with the assistance of a soluble organic-inorganic composite film, which accelerated the photocatalytic process two-fold compared to the unpoled BiFeO_3_ [36].

In this work, as an extension to the research on the performance improvement of NBT catalysts, NBT powder was prepared using the hydrothermal method, and a technique called high-voltage poling was employed to enhance its built-in electric field. The effect and mechanism of the poling process on the catalytic performance of NBT were discussed. It was found that the poling-enhanced built-in electric field can provide a strong driving force for the separation of photogenerated carriers. The degradation of RhB solution can reach 100% within 120 min, and the reaction rate constant *k*_obs_ is 28.36 × 10^−3^ min^−1^, which is much higher than that of unpoled NBT.

## 2. Experimental Section

### 2.1. Preparation of Catalysts

The NBT catalyst was prepared by a typical hydrothermal method. Firstly, 2.1828 g Bi(NO_3_)_3_·5H_2_O (99%, Shanghai Macklin Biochemical Co., Shanghai, China) and 0.0410 g CH_3_COONa (99%, Fuchen Chemical Reagent Co., Tianjin, China) were dissolved in 20 mL deionized water and stirred for 30 min. Then, 1.3 mL Ti(C_4_H_9_O)_4_ (98%, Fuchen Chemical Reagent Co., Tianjin, China) was added slowly and followed by continuous stirring for 30 min. Subsequently, 40 mL NaOH (96%, Fuchen Chemical Reagent Co., Tianjin, China) solution was slowly poured into the above solution to make the concentration of NaOH in the solution reach 3 M. After stirring for 120 min, the mixture was transferred into a 150 mL Teflon autoclave and heated at 180 °C for 20 h. At last, the samples were washed 3 times with ethanol and deionized water and then dried at 80 °C.

A 0.6 g amount of NBT was weighed and pressed at 150 MPa for 2 min to obtain a disc, and conductive silver paste was coated on both sides of the disc. Then, samples were poled under DC voltage in a silicone oil medium. The poling electric field was set to 0 kV mm^−1^, 1 kV mm^−1^, and 2 kV mm^−1^ for 30 min. After poling, the silver electrode on the surface of the sample was ground with 2000-mesh sandpaper, and the disc was broken with a mortar. Subsequently, the powder catalysts were ball-milled for 4 h in ethanol and dried at 100 °C to obtain the catalyst. According to the different poling electric fields, the samples were named unpoled NBT, 1 kV mm^−1^ NBT, and 2 kV mm^−1^ NBT, respectively.

### 2.2. Characterization

X-ray diffraction (XRD) was performed by AXS D8 Advance (Cu Kα radiation source, λ = 1.5406 Å) to characterize the phase structure of the catalysts. A scanning electron microscope (SEM, Hitachi S4800, Tokyo, Japan) was used to characterize the microstructure of the catalysts. The light absorption performance was characterized by ultraviolet–visible diffuse reflectance spectrum (UV–VIS DRS, Hitachi UH-4150, Tokyo, Japan). The photoluminescence (PL) spectrum was measured by FLS-1000 and the excitation wavelength was 300 nm. Electrochemical properties were characterized by an electrochemical workstation (CHI660E Instruments, Shanghai, China).

The photocatalytic and piezo-photocatalytic activities of NBT were evaluated by degrading Rhodamine B (RhB, 5 mg/L) dye solutions, utilizing a 300 W Xenon lamp as the light source (L) and magnetic stirrer (S) as the external force. During the catalytic process, 100 mg NBT was added to 100 mL RhB solution and stirred in the dark for 30 min to achieve adsorption-desorption equilibrium. Subsequently, 2 mL of suspension was extracted every 20 min and centrifuged to remove the catalyst particles. The removal of RhB was tested based on the absorption at 554 nm by a UV–VIS spectrophotometer. The degradation rate of RhB can be calculated by (*C*_0_ − *C*_t_)/*C*_0_, where *C*_0_ and *C*_t_ are the initial concentration and residual concentration of the solution, respectively.

To reveal the active species involved in the catalytic process, disodium ethylenediaminetetraacetate (EDTA-2Na), isopropyl alcohol (IPA), and benzoquinone (BQ) were added to the RhB solution as scavengers of holes (h^+^), hydroxyl radicals (·OH) and superoxide radicals ($O_{2}^{-}$), respectively. The subsequent procedure was similar to the catalytic degradation experiments described above.

## 3. Results and Discussion

**Figure 1:**
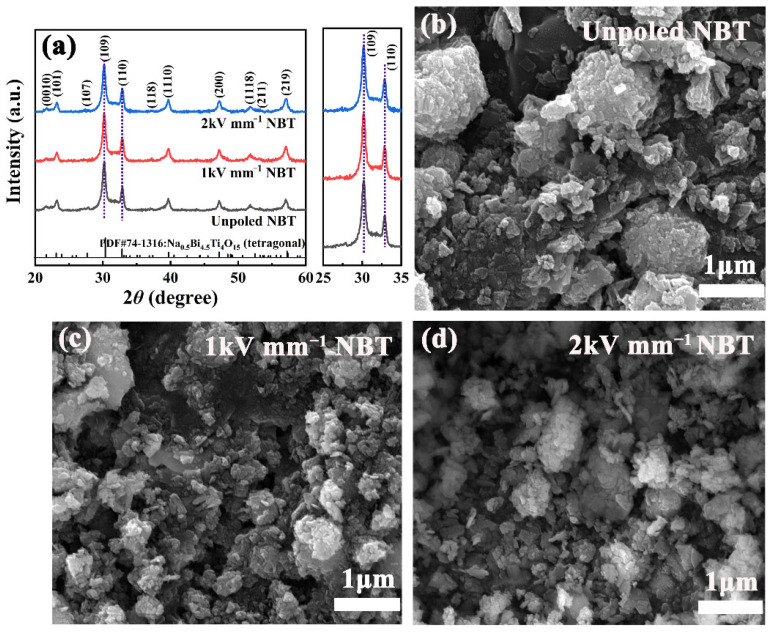
(**a**) XRD patterns and (**b**–**d**) SEM images of the unpoled NBT, 1 kV mm^−1^ NBT, and 2 kV mm^−1^ NBT samples.

Figure 1a shows the XRD patterns of as-prepared unpoled NBT, 1 kV mm^−1^ NBT, and 2 kV mm^−1^ NBT. It can be seen that all diffraction peaks are derived from the Na_0.5_Bi_4.5_Ti_4_O_15_ phase (JCPDS#74-1316), and no obvious secondary phase was traced. In addition, the intensity ratio of (110) and (109) increases from 0.4871 in the unpoled NBT sample to 0.5254 in the 2 kV mm^−1^ NBT samples, which results from the high field poling. Moreover, SEM images of the unpoled NBT, 1 kV mm^−1^ NBT, and 2 kV mm^−1^ NBT are plotted in Figure 1b–d. As can be seen, the morphologies of the unpoled NBT, 1 kV mm^−1^ NBT, and 2 kV mm^−1^ NBT samples are quite similar.

**Figure 2:**
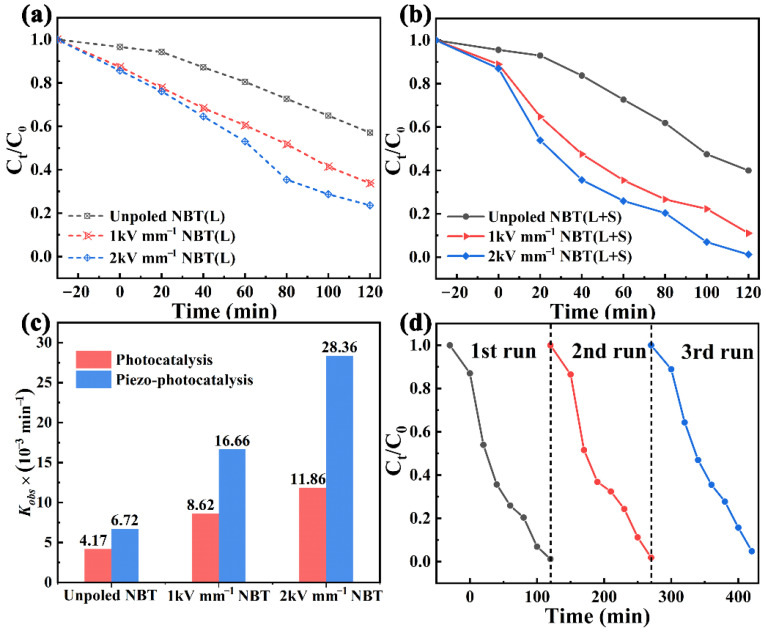
The degradation of RhB for the unpoled and poled NBT: (**a**) photocatalysis and (**b**) piezo-photocatalysis; (**c**) *k*_obs_ of the unpoled and poled NBT under photocatalysis and piezo-photocatalysis; (**d**) recycling experiment of the 2 kV mm^−1^ NBT for piezo-photocatalysis of RhB.

To evaluate the catalytic activity and explore the effect of the high-voltage poling process on photocatalysis and piezo-photocatalysis, the degradation of RhB solution by unpoled and poled NBT was investigated. The photocatalytic ability was evaluated under light irradiation within 120 min, as featured in Figure 2a. The RhB degradation efficiencies reached 42.9%, 66.2%, and 76.4%, respectively, for the unpoled NBT, 1 kV mm^−1^ NBT, and 2 kV mm^−1^ NBT. In contrast, the poled NBT revealed superior catalytic degradation activity with the increase in applied poling electric field, indicating that the introduction of a poling electric field can provide a driving force for the separation and transfer of photogenerated carriers to result in an evident enhancement of photocatalytic activity [37]. Interestingly, when stirring and simulated sunlight irradiations were simultaneously applied to the RhB solution, a remarkable improvement could be observed in the piezo-photocatalysis for each of these samples compared with that of individual photocatalysis (see Figure 2b). In addition, with the increase in the poling electric field, the photocatalytic and piezo-photocatalytic properties are significantly enhanced. For the 2 kV mm^−1^ NBT sample, its piezo-photocatalytic efficiency (100%) was about 11.0% higher than that of the 1 kV mm^−1^ NBT and 40% higher than that of the unpoled NBT, confirming that the enhancement of poling voltage can strengthen the built-in electric field and promote photocatalytic and piezo-photocatalytic reaction process.

The kinetics of photocatalytic and piezo-photocatalytic activities of NBT may follow a first-order reaction [38,39]:

(1) $$\ln(\frac{C_{0}}{C_{t}})=k_{obs}t$$

where *C*_0_ and *C*_t_ are the initial and residual concentrations at reaction time *t*, and *k*_obs_ is the observed pseudo-first-order reaction rate constant (min^−1^). As shown in Figure 2c, the *k*_obs_ value can be obtained from the slope of (ln*C*_0_/*C*_t_) vs. *t*. It can be seen that the *k*_obs_ of 2 kV mm^−1^ NBT under piezo-photocatalysis can reach 28.36 × 10^−3^ min^−1^, which is ~2.4 times larger than that of 2 kV mm^−1^ NBT under single photocatalysis (11.86 × 10^−3^ min^−1^), ~1.7 times larger than that of 1 kV mm^−1^ NBT under piezo-photocatalysis (16.66 × 10^−3^ min^−1^), ~4.2 times larger than that of unpoled NBT under piezo-photocatalysis (6.72 × 10^−3^ min^−1^), and even ~6.8 times larger than that of unpoled NBT under single photocatalysis (4.17 × 10^−3^ min^−1^). The above results further indicate that the introduction of a poling electric field can enhance the photocatalytic and piezo-photocatalytic performance of NBT, and the improvement of performance is closely related to the intensity of the poling electric field. To highlight the catalytic activity of the 2 kV mm^−1^ NBT, we summarized the degradation rate and *k*_obs_ values of recently reported piezo-photocatalysts, as shown in Table 1. It can be seen that the *k*_obs_ value of 2 kV mm^−1^ NBT is superior to that of most reported piezo-photocatalysts such as BiFeO_3_, Na_0.5_Bi_0.5_TiO_3_ and BiVO_4_ [7,10,40], which indicates that this work provides a choice and idea for the development and utilization of other high-performance bismuth layer structure catalysts. The reproducibility and stability of catalysts are crucial for practical application. Herein, three consecutive cycles are conducted on the 2 kV mm^−1^ NBT by adding a fresh RhB solution directly after the vaporization of the previous solution, and the results are compared in Figure 2d. The degradation rate of RhB solution does not decrease significantly after three cycles, implying that the 2 kV mm^−1^ NBT possesses excellent piezo-photocatalytic stability and recyclability.

**Figure 3:**
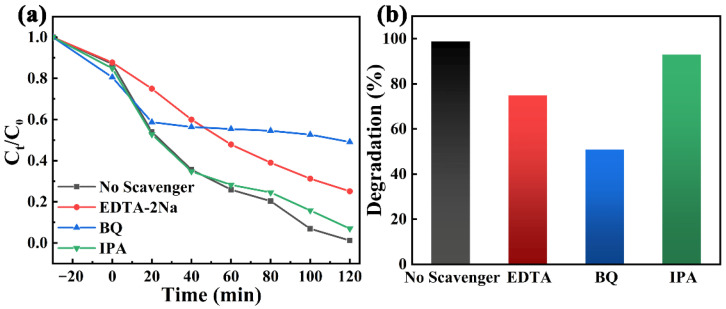
(**a**) Free-radical trapping experiments of the 2 kV mm^−1^ NBT under piezo-photocatalysis; (**b**) corresponding degradation rates at 120 min.

To determine the active species in the piezo-photocatalytic process of the 2 kV mm^−1^ NBT, free radical capture experiments were carried out, and EDTA-2Na, BQ, and IPA were used as capture agents for h^+^, ·$O_{2}^{-}$, and ·OH, respectively, as shown in Figure 3a,b. The degradation of the RhB solution was inhibited after the addition of BQ and EDTA-2Na, and the degradation rates were reduced to 50.93% and 74.89%, respectively. However, the addition of IPA only decreased the degradation rate from 100% to 93%, indicating that the main active species in the process of 2 kV mm^−1^ NBT piezo-photocatalytic are ·$O_{2}^{-}$ and h^+^.

**Figure 4:**
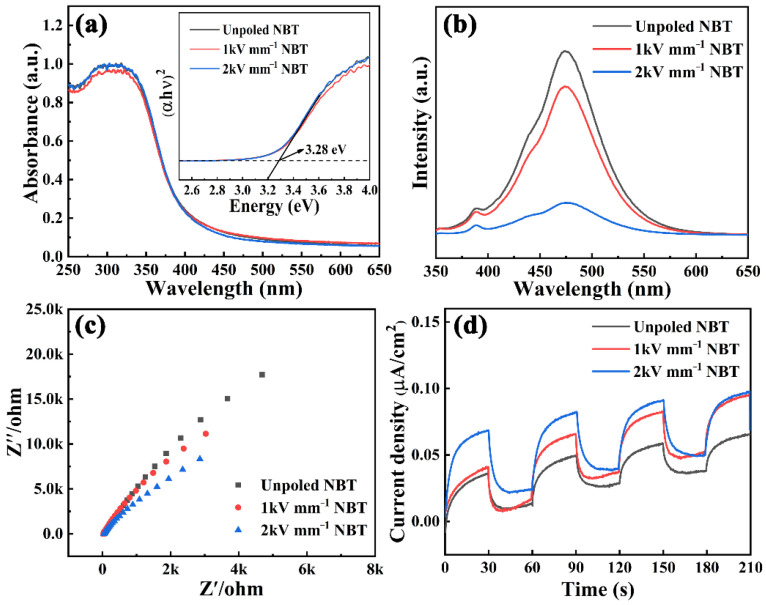
(**a**) UV–VIS DRS, (**b**) PL spectra, (**c**) electrochemical impedance spectroscopy, and (**d**) photocurrent–time response curves of the unpoled and poled NBT.

In order to explore the effect of the poling process on the light absorption performance, the UV–VIS DRS was characterized on the unpoled and poled NBT. As illustrated in Figure 4a, the optical absorption edge is estimated to be at 405 nm, which determines that NBT is a semiconductor catalyst for ultraviolet excitation. In addition, the increase in poling electric field will not affect the light absorption intensity and band gap width of the NBT, and the high piezo-photocatalytic activity of the 2 kV mm^−1^ NBT is independent of the light response. It is well known that the separation and transfer efficiency of photogenerated carriers is the key to determining the photocatalytic performance [18,41]. Consequently, photoluminescence spectroscopy (PL), electrochemical impedance spectroscopy (EIS), and transient photocurrent response tests were performed on the unpoled and poled NBT to further investigate the enhanced mechanism of poling on photocatalysis. As plotted in Figure 4b, the decreasing order of the fluorescence intensity is 2 kV mm^−1^ NBT < 1 kV mm^−1^ NBT < unpoled NBT, and a lower fluorescence intensity means a low recombination rate of electron–hole pairs [11,19,42,43], which indicates that the application of poling electric field can hinder the recombination of photogenerated charges. In addition, the larger the poling electric field, the lower the fluorescence intensity and the higher the catalytic activity. The EIS curves are shown in Figure 4c, and it was found that the arc radius of the 2 kV mm^−1^ NBT is the smallest, while the unpoled NBT has the largest arc radius. Generally, the smaller the arc radius in the EIS Nyquist plot, the lower the charge transfer resistance will be [44,45], which confirms that the poling process can promote the transfer of photogenerated carriers. As well known, the higher photocurrent response means a higher charge carrier density and efficient charge carrier separation, which is beneficial to photocatalytic performance [46]. In Figure 4d, the 2 kV mm^−1^ NBT sample has the highest photocurrent density, further verifying the high catalytic activity of 2 kV mm^−1^ NBT is due to the poled-enhanced built-in electric field [37].

**Figure 5:**
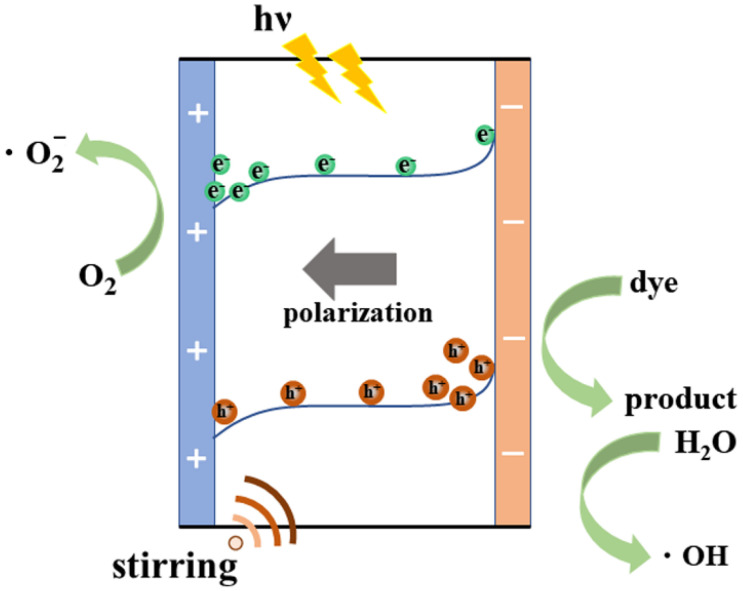
Piezo-photocatalysis mechanism of the poled NBT.

Figure 5 presents the enhanced mechanism of poled NBT piezo-photocatalysis. Firstly, NBT is excited by light to produce electrons and holes, which occupy the conduction band (CB) and valence band (VB) of the material, respectively. Simultaneously, due to the existence of stirring, NBT will generate an internal electric field. The generation of a built-in electric field can not only provide a driving force for the separation and transfer of photogenerated electrons and holes, but also lead to the accumulation of positive and negative charges on the two relative surfaces of NBT, which effectively inhibits the recombination of photogenerated carriers [47]. When a poling electric field is applied to NBT, the ferroelectric domains of NBT tend to be ordered, and the polarization direction tends to be consistent, which effectively enhances the built-in electric field of NBT and improves the separation efficiency and lifetime of photogenerated carriers [34,48].

## 4. Conclusions

In summary, we successfully synthesized the NBT catalyst using the hydrothermal method and optimized its piezo-photocatalytic performance through high-voltage poling. The as-prepared 2 kV mm^−1^ NBT sample exhibited the higher piezo-photodegradation capability for RhB, the decolorization rate of RhB solution reaches 100% within 120 min, and the reaction rate constant *k*_obs_ reaches 28.36 × 10^−3^ min^−1^, which is much higher than that of the unpoled NBT. The enhanced photo-piezocatalytic performance of the 2 kV mm^−1^ NBT was attributed to the poling-enhanced internal electric field, which can effectively promote the separation and transfer of photogenerated carriers. In addition, recycling experiments proved that the 2 kV mm^−1^ NBT is an effective and reusable material for dye degradation. Our work may provide a novel approach for the exploration and development of highly efficient semiconductor piezo-photocatalysts.

## Figures and Tables

**Table 1:** Degradation rate and *k*_obs_ values of the recently reported piezo-photocatalysts.

Piezo-Photocatalyst	Pollution	Degradation Rate (Rate (%)-Time)	*k*_obs_ × 10^−3^ (min^−1^)	Reference
BiFeO_3_/TiO_2_	MV (10 mg/L)	100%—120 min	24.0	[40]
BiFeO_3_	MB (20 mg/L)	80%—90 min	-	[10]
Na_0.5_Bi_0.5_TiO_3_	RhB (10 mg/L)	63.6%—60 min	16.4	[7]
BiVO_4_	RhB (10 mg/L)	58.0%—60 min	14.3	[7]
2 kV mm^−1^ NBT	RhB (10 mg/L)	100%—120 min	28.4	This work

## Data Availability

The data presented in this study are available on request from the corresponding author.
